# Uso de Stent Recoberto Manualmente com Tegaderm em Perfurações Vasculares: Relato de Série de Casos

**DOI:** 10.36660/abc.20250834

**Published:** 2026-07-28

**Authors:** Alexandre Damiani Azmus, Antônio Carlo Klug Cogo, Luis Manuel Rodriguez Oyuela, João Vitor Slaviero, Andre Luiz Langer Manica, Eduardo Mascarenhas Azevedo

**Affiliations:** 1 Instituto de Cardiologia Porto Alegre RS Brasil Instituto de Cardiologia, Porto Alegre, RS – Brasil

**Keywords:** Stents, Intervenção Coronária Percutânea, Relatos de Caso

## Introdução

A perfuração coronariana durante intervenção coronariana percutânea (ICP) representa uma complicação rara, porém potencialmente letal, cuja incidência é estimada em 0,2% a 0,6% das angioplastias.^1^

Os fatores de risco incluem idade avançada, cirurgia de revascularização miocárdica (CRM) prévia, doença coronariana complexa, tortuosidade, calcificação ou presença de oclusões totais crônicas.^2^ A classificação de Ellis permanece amplamente utilizada para definir gravidade e orientar conduta.^3^

As estratégias terapêuticas estabelecidas incluem insuflação prolongada de balão, reversão de anticoagulação, embolização distal, drenagem pericárdica em caso de tamponamento e implante de stents recobertos.^4^ Contudo, em centros de saúde pública, o acesso a stents recobertos industriais pode ser limitado pelo custo, tornando necessária a adoção de soluções emergenciais.

Neste contexto, o uso de stent recoberto manualmente com filme Tegaderm surge como opção de *bailout* extremo em casos de indisponibilidade das estratégias *on-label*.^5–7^ O presente trabalho descreve três casos tratados em um hospital do Rio Grande do Sul, nos quais a técnica foi empregada para controlar perfurações vasculares.

## Casos clínicos

### Caso 1

Homem, 64 anos, com CRM prévia, submetido à ICP da artéria coronária direita (ACD). Após conclusão do procedimento, identificado perfuração distal de subramos da artéria descendente posterior (ADP) pela ponta do fio-guia (utilizados Whisper e BHW) (figura 1). Foi realizado insuflação de balão por 40 minutos, sem resolução. Realizado implante de stent recoberto PK Papyrus 2,5 x 20 mm com 8 ATMs sem resolução do extravasamento. Devido a indisponibilidade de outro stent recoberto, foi realizado o implante de stent Orsiro 2,5 x 22mm recoberto manualmente com Tegaderm com 14 ATMs. A angiografia final demonstrou interrupção do extravasamento e fluxo TIMI 3, apesar da expansão irregular do stent (provavelmente pela cobertura com Tegaderm). O paciente recebeu alta assintomático e sem complicações.

**Figura 1:**
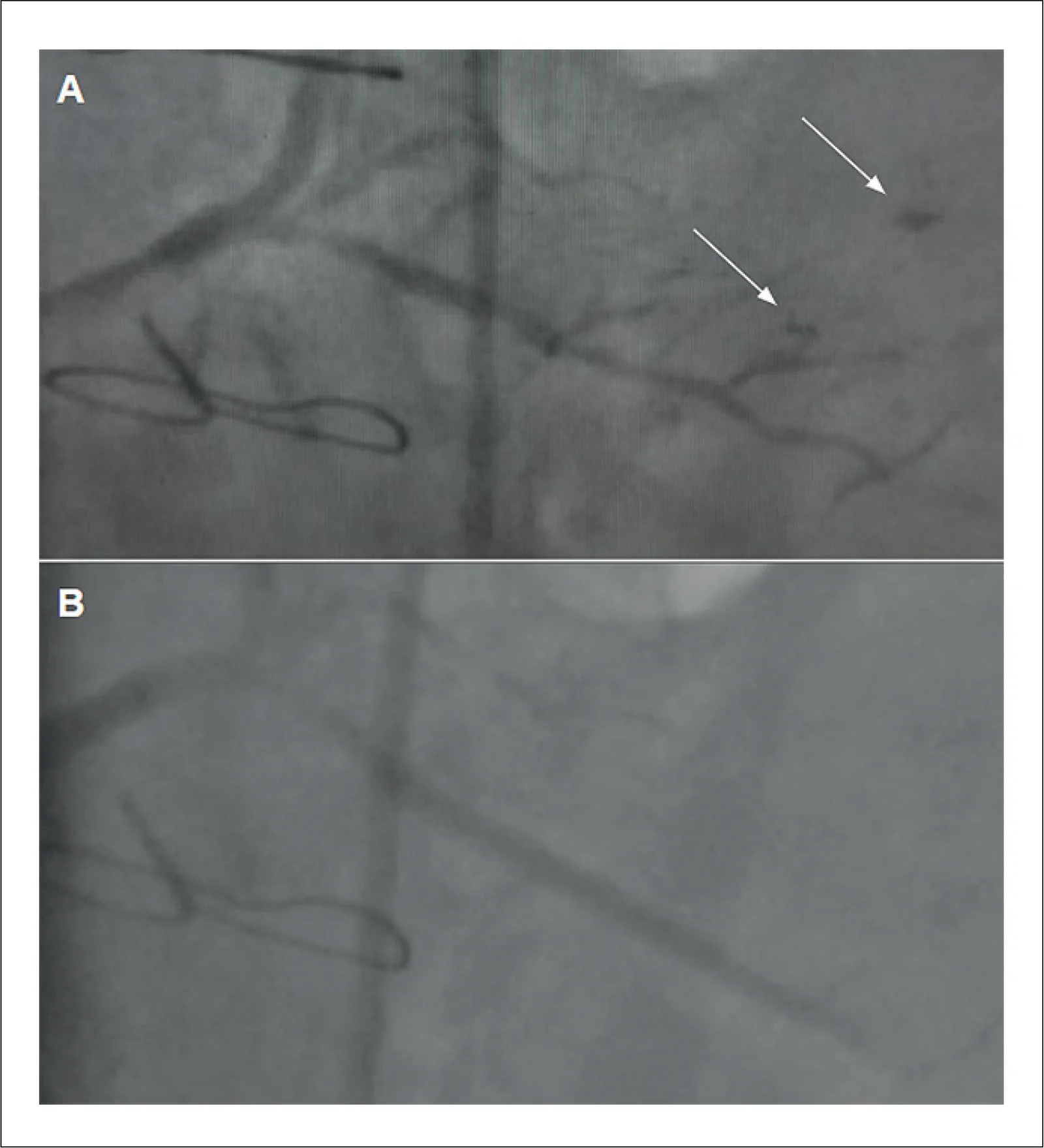
A) Perfuração de subramos da artéria descendente posterior tipo Ellis III (seta branca), onde se observa extravasamento de contraste. B) Resultado final após implante de stent recoberto com Tegaderm com melhora do extravasamento do contraste.

### Caso 2

Homem, 64 anos, submetido à ICP de Artéria Descendente Anterior (ADA) e ramo diagonal. Após pré-dilatação do ramo diagonal com balão NC Trek Neo 2,5x20mm com 18 ATMs se observou perfuração tipo Ellis III (figura 2). Realizada insuflação do balão por 20 minutos, sem sucesso. Foi realizado implante de stent SupraFlex 2,5 x 28mm recoberto com Tegaderm com 12 ATMs. A angiografia final demonstrou interrupção do extravasamento e fluxo TIMI 3. O paciente recebeu alta assintomático e sem complicações.

**Figura 2:**
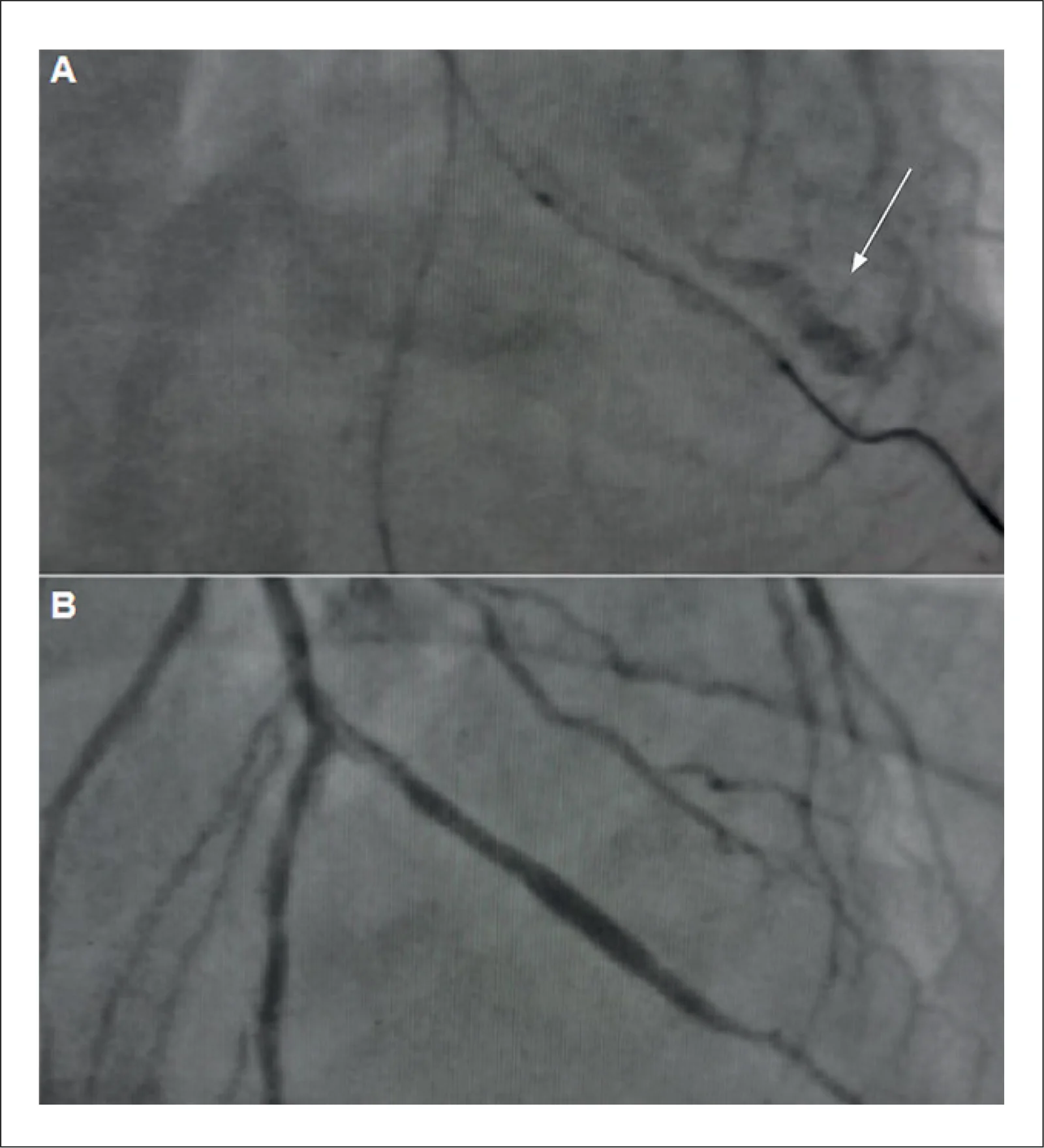
A) Perfuração de ramo diagonal tipo Ellis III com extravasamento de contraste (seta branca). B) Resultado final após implante de stent recoberto com Tegaderm com melhora do extravasamento de contraste.

### Caso 3

Homem, 76 anos, submetido a ICP da ACD via acesso radial direito. Durante o procedimento, ocorreu perfuração da artéria radial acessória pela passagem do fio-guia 0,035", com formação subsequente de pseudoaneurisma (figura 3). Realizadas inúmeras tentativas de compressão local guiada por ecografia, sem sucesso. Foi optado por implante de stent Xience 4,0 x 33mm recoberto com Tegaderm com 15 ATMs na artéria radial. A angiografia final demonstrou ausência de extravasamento e preservação do fluxo do membro. O paciente apresentou boa evolução e teve alta hospitalar assintomático e sem complicações.

**Figura 3:**
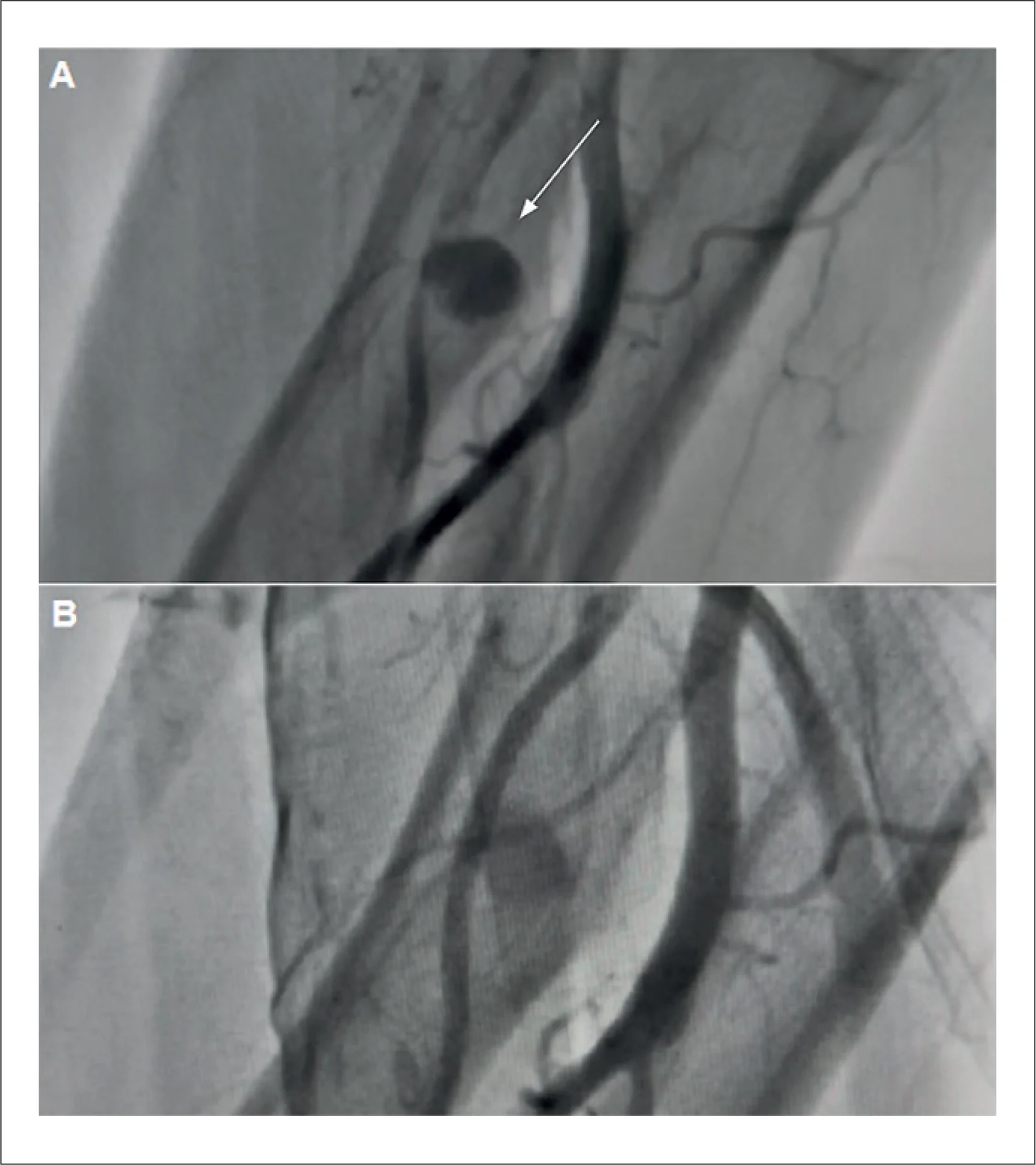
A) Perfuração de artéria radial acessória com extravasamento de contraste e formação de pseudoaneurisma (seta branca). B) Resultado final com melhora do extravasamento de contraste, restando apenas blush de contraste aprisionado em tecido subcutâneo.

## Discussão

A perfuração coronariana é uma complicação grave, com mortalidade de até 20% dos casos.^8^ O manejo inicial deve priorizar a estabilidade hemodinâmica e o tamponamento da lesão com insuflação de balão por tempo prolongado. Persistindo o extravasamento, o implante de stent recoberto é um dos tratamentos recomendados.^4,8^ Entretanto, na indisponibilidade deste material, especialmente em centros de saúde pública de países em desenvolvimento, pode se fazer necessária a adoção de soluções alternativas.

Relatos recentes demonstram que o stent recoberto manualmente com Tegaderm pode ser eficaz no selamento de perfurações coronárias, com sucesso técnico imediato em mais de 90% dos casos.^5–7^ Nos três casos relatados houve interrupção do extravasamento de contraste, sem complicações durante a internação hospitalar (Tabela 1). A técnica baseia-se na oclusão direta da parede vascular. Além disso, o uso da técnica na artéria radial ilustra, adicionalmente, a aplicabilidade periférica da técnica. Não foi utilizado método de imagem intravascular por indisponibilidade no sistema público de saúde.

**Tabela 1:** Características clínicas e angiográficas dos casos clínicos relatados

Caso	Idade	Sexo	Quadro clínico	Stent Recoberto com Tegaderm^®^
1	64 anos	Masculino	Perfuração (Ellis 3) de subramos da ADP pela ponta do fio guia após ICP da ACD.	Orsiro 2,5x22mm
2	64 anos	Masculino	Perfuração da artéria diagonal (Ellis 3) após pré-dilatação com balão NC.	SupraFlex 2,5x28mm
3	76 anos	Masculino	Perfuração com formação de pseudoaneurisma da artéria radial acessória causado pela passagem do fio-guia 0,035" durante ICP.	Xience 4,0x33mm

O Tegaderm, apesar de desenvolvido para uso tópico, é composto de poliuretano, o mesmo material já utilizado nos stents recobertos industriais, porém com densidades e processamentos químicos diferentes. Nos casos relatados o stent foi recoberto com duas voltas completas do Tegaderm, deixando-se uma distância de aproximadamente 1 mm das bordas da estrutura metálica, conforme recomendado (Figura 4).^5^

**Figura 4:**
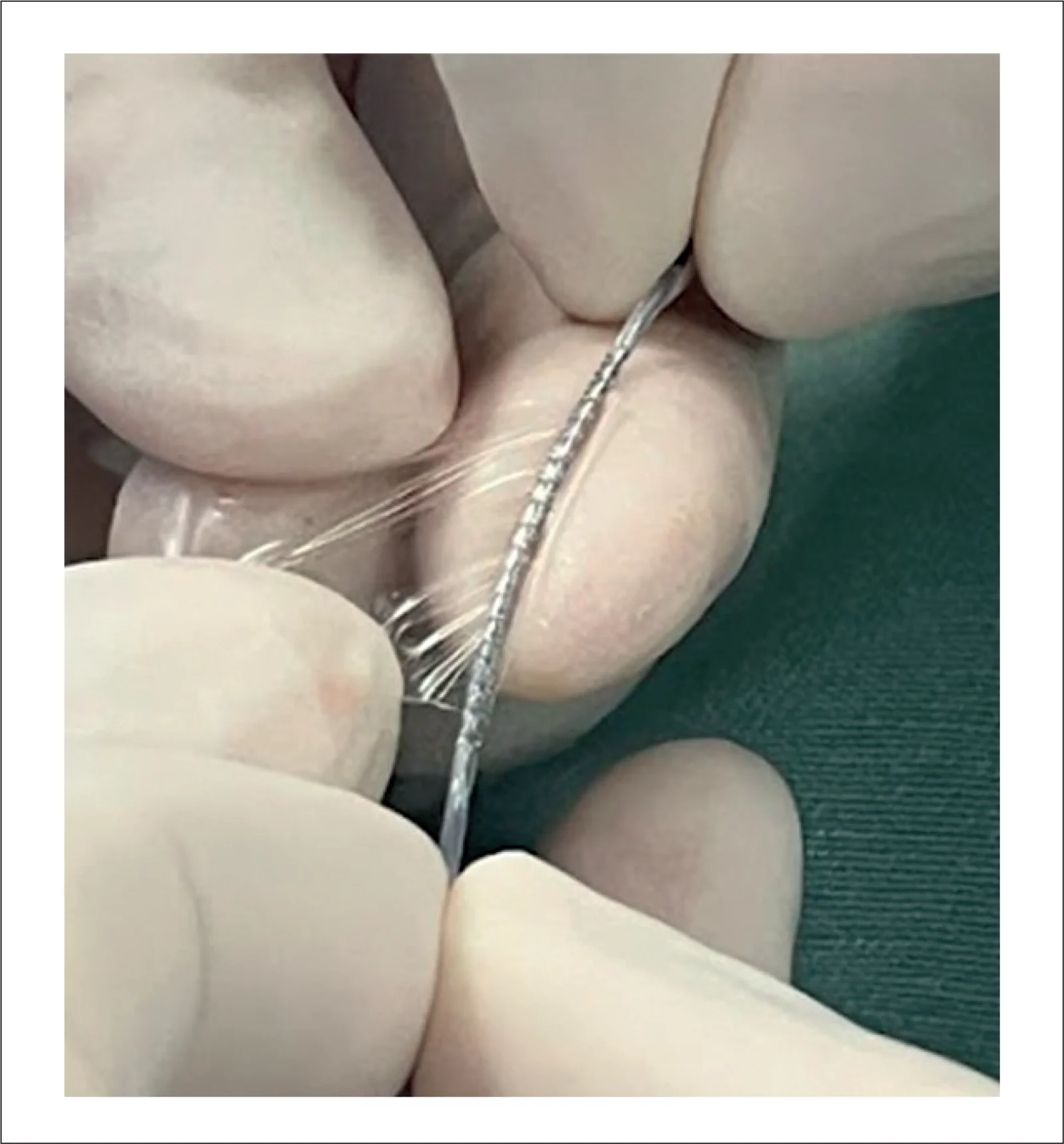
Recobrimento manual de stent com Tegaderm.

As principais limitações dessa técnica são o maior risco de trombose e reestenose em comparação aos stents recobertos industriais.^9,10^ A técnica com Tegaderm apresenta desvantagens estruturais: ausência de camada de adesão uniforme, potencial rugosidade interna e ausência de validação regulatória. Portanto, deve ser usado sobretudo em situações de risco à vida como "bailout extremo" em que não há outra opção terapêutica *"on-label"* disponível. Portanto, é fundamental manter dupla antiagregação plaquetária e acompanhamento clínico rigoroso.

## Conclusão

A perfuração coronariana durante ICP é uma complicação rara e potencialmente fatal. O uso emergencial de stent recoberto com Tegaderm pode ser eficaz no controle imediato do extravasamento e na preservação do fluxo vascular, constituindo alternativa possível como "*bailout* extremo" em contexto de risco iminente de vida e limitação absoluta de outras terapias já estabelecidas. É fundamental ressaltar que a técnica constitui estratégia *off-label*, não existindo ensaios clínicos ou estudos prospectivos que avaliem a segurança e a eficácia dessa técnica.

## Data Availability

Os conteúdos subjacentes ao texto da pesquisa estão contidos no manuscrito.
