# Supplementary material for: Uso de Stent Recoberto Manualmente com Tegaderm em Perfurações Vasculares: Relato de Série de Casos
Source: Arq Bras Cardiol. 2026 Jul 28;123(8):e20250834. [Article in Portuguese] doi: 10.36660/abc.20250834 (PMC13649064; doi:10.36660/abc.20250834)
Supplement: Translation

# Translation: Use of Manually Covered Stent with Tegaderm in Vascular Perforations: Case Series Report

**DOI:** 10.36660/abc.20250834i

**Authors:** Alexandre Damiani Azmus, Antônio Carlo Klug Cogo, Luis Manuel Rodriguez Oyuela, João Vitor Slaviero, Andre Luiz Langer Manica, Eduardo Mascarenhas Azevedo

**Affiliations:** 1 Porto Alegre RS Brazil Instituto de Cardiologia, Porto Alegre, RS – Brazil

**Keywords:** Stents, Intervenção Coronária Percutânea, Case Reports

## Introduction

Coronary perforation during percutaneous coronary intervention (PCI) is a rare but potentially lethal complication, with an estimated incidence of 0.2% to 0.6% of angioplasties.^1^

Risk factors include advanced age, previous coronary artery bypass graft (CABG) surgery, complex coronary artery disease, tortuosity, calcification, or the presence of chronic total occlusions.^2^ The Ellis classification remains widely used to define severity and guide management.^3^

Established therapeutic strategies include prolonged balloon inflation, reversal of anticoagulation, distal embolization, pericardial drainage in cases of tamponade, and implantation of covered stents.^4^ However, in public health centers, access to industrial covered stents may be limited by cost, making it necessary to adopt emergency solutions.

In this context, the use of manually coated stents with Tegaderm film emerges as an extreme bailout option in cases where on-label strategies are unavailable.^5–7^ This paper describes three cases treated at a hospital in Rio Grande do Sul, in which the technique was used to control vascular perforations.

## Clinical cases

### Case 1

A 64-year-old man with a history of coronary artery disease underwent PCI for right coronary artery bypass grafting (RCA). (RCABG). After completion of the procedure, distal perforation of sub-branches of the posterior descending artery (PDA) was identified by the tip of the guidewire (Whisper and BHW were used) (Figure 1). Balloon inflation was performed for 40 minutes, without resolution. A 2.5 x 20 mm PK Papyrus covered stent with 8 ATMs was implanted, without resolution of the leakage. Due to the unavailability of another covered stent, a 2.5 x 22 mm Orsiro stent manually covered with Tegaderm with 14 ATMs was implanted. Final angiography demonstrated interruption of leakage and TIMI 3 flow, despite irregular stent expansion (probably due to the Tegaderm covering). The patient was discharged asymptomatic and without complications.

**Figure 1:** A) Perforation of sub-branches of the Ellis III type posterior descending artery (white arrow), showing contrast extravasation. B) Final result after implantation of a Tegaderm-coated stent with improvement in contrast extravasation.

### Case 2

A 64-year-old man underwent PCI of the left anterior descending artery (LAD) and diagonal branch. After pre-dilation of the diagonal branch with a 2.5x20mm NC Trek Neo balloon with 18 ATMs, an Ellis III type perforation was observed (Figure 2). Balloon inflation was performed for 20 minutes without success. A 2.5 x 28mm SupraFlex stent covered with Tegaderm was implanted with 12 ATMs. Final angiography demonstrated interruption of extravasation and TIMI 3 flow. The patient was discharged asymptomatic and without complications.

**Figure 3:** A) Perforation of an accessory radial artery with contrast extravasation and pseudoaneurysm formation (white arrow). B) Final result with improved contrast extravasation, leaving only contrast blush trapped in subcutaneous tissue.

### Case 3

A 76-year-old man underwent PCI of the RCA via a right radial approach. During the procedure, perforation of the accessory radial artery occurred due to the passage of the 0.035" guidewire, with subsequent formation of a pseudoaneurysm (Figure 3). Numerous attempts at local compression guided by ultrasound were unsuccessful. A 4.0 x 33 mm Xience stent covered with Tegaderm with 15 ATMs was implanted in the radial artery. Final angiography showed no leakage and preserved limb flow. The patient had a good recovery and was discharged asymptomatic and without complications.

**Figure 2:** A) Diagonal branch perforation type Ellis III with contrast extravasation (white arrow). B) Final result after implantation of a Tegaderm-coated stent with improvement in contrast extravasation.

## Discussion

Coronary perforation is a serious complication, with mortality rates up to 20%.^8^ Initial management should prioritize hemodynamic stability and tamponade of the lesion with prolonged balloon inflation. If leakage persists, implantation of a covered stent is one of the recommended treatments.^4,8^ However, in the unavailability of this material, especially in public health centers in developing countries, alternative solutions may be necessary.

Recent reports demonstrate that manually coated stents with Tegaderm can be effective in sealing coronary perforations, with immediate technical success in over 90% of cases.^5–7^ In the three reported cases, contrast extravasation was stopped without complications during hospitalization (Table 1). The technique is based on direct occlusion of the vascular wall. Furthermore, the use of the technique in the radial artery further illustrates the peripheral applicability of the technique. Intravascular imaging was not used due to its unavailability in the public health system.

**Table 1:** Clinical and angiographic characteristics of the reported clinical cases

Case	Age	Sex	Clinical picture	Stent coated with Tegaderm^®^
1	64 years old	Masculine	Perforation (Ellis 3) of PDA subbranches by the guidewire tip after RCA PCI.	Orsiro 2.5x22mm
2	64 years old	Masculine	Diagonal artery perforation (Ellis 3) after pre-dilation with NC balloon.	SupraFlex 2.5x28mm
3	76 years old	Masculine	Perforation with pseudoaneurysm formation of the accessory radial artery caused by the passage of a 0.035" guidewire during PCI.	Xience 4.0x33mm

Tegaderm, although developed for topical use, is composed of polyurethane, the same material already used in industrial covered stents, but with different densities and chemical processing. In the reported cases, the stent was covered with two complete turns of Tegaderm, leaving a distance of approximately 1 mm from the edges of the metallic structure, as recommended (Figure 4).^5^

**Figure 4:** Manual application of Tegaderm to a stent.

The main limitations of this technique are the higher risk of thrombosis and restenosis compared to industrial covered stents.^9,10^ The Tegaderm technique has structural disadvantages: lack of a uniform adhesion layer, potential internal roughness, and lack of regulatory validation. Therefore, it should be used primarily in life-threatening situations such as "extreme bailout" where no other "on-label" therapeutic option is available. Therefore, it is essential to maintain dual antiplatelet therapy and rigorous clinical monitoring.

## Conclusion

Coronary perforation during PCI is a rare and potentially fatal complication. The emergency use of a Tegaderm-coated stent can be effective in immediately controlling extravasation and preserving vascular flow, constituting a possible alternative as an "extreme bailout" in contexts of imminent risk of death and absolute limitation of other established therapies. It is crucial to emphasize that this technique constitutes an off-label strategy, and there are no clinical trials or prospective studies evaluating the safety and efficacy of this technique.

## Data Availability

The underlying content of the research text is contained within the manuscript.
